# Health-related quality of life in type-2 diabetes patients: a cross-sectional study in East China

**DOI:** 10.1186/s12902-017-0187-1

**Published:** 2017-07-06

**Authors:** You Lu, Ningjian Wang, Yi Chen, Xiaomin Nie, Qin Li, Bing Han, Yingchao Chen, Fangzhen Xia, Zhen Cang, Meng Lu, Ying Meng, Yingli Lu

**Affiliations:** Institute and Department of Endocrinology and Metabolism, Shanghai Ninth People’s Hospital Affiliated to Shanghai Jiaotong University School of Medicine, Shanghai, 200011 China

**Keywords:** EQ-5D, HRQoL, Diabetes, East China

## Abstract

**Background:**

Used the EuroQoL-5 dimension (EQ-5D) to evaluate the health status of 5310 residents who live in East China, and compared the health-related quality of life (HRQoL) with 311 patients with type 2 diabetes as well as to explore the main influence factors to HRQoL in East China.

**Methods:**

The cohort includes 5310 participants aged 18-89 years old lived in East China. EuroQoL-5 dimension (EQ-5D) scale was used for the assessment of health-related quality of life.

**Results:**

The mean age of the cohort was 52.2 ± 13.4 years of which 43.7% were male. A moderate level of health-related quality of life was measured of that EQ-5D index and EQ-VAS scores were 0.939 ± 0.111 and 80.06 ± 11.58, respectively. There was a significant difference between diabetes patients and non-diabetes (*p* = 0.029, *p* < 0.001, respectively). The age had an inverse correlation with the EQ-5D scores both in general population and diabetes patients. The EQ-5D Vas was weakly adversely associated with the FPG, HbA1c and HOMA-IR.

**Conclusion:**

The overall health-related quality of life of population in East-China was moderate. Diabetes patients had lower score of health-related quality. The healthy-related quality was associated with the age, gender, economic development of region, level of education and marital status.

## Background

The prevalence of diabetes mellitus (DM) has been increasing significantly in recent decades. It is now reaching epidemic proportions worldwide [1, 2]. In China, the age-standardized prevalence of adult diabetes and prediabetes were 11.6 and 50.1%, respectively, accounting for 113.9 million people with diabetes and 493.4 people million with prediabetes in 2013 [3]. The International Diabetes Federation (IDF) has estimated that approximately 592 million adults in worldwide will have diabetes in 2035 [4]. Diabetes has an adverse effect on quality of life. Most patients suffer from a variety of long-term complications including micro-vascular complications (e.g. neuropathy, nephrolopathy and retinopathy) and macro-vascular complications (e.g. myocardial infarction, angina pectoris, stroke and amputation) [5]. Besides, the trouble of taking oral antidiabetic agents several times a day, the fear of subcutaneous injection of insulin, and incidents of hypoglycemia might depress diabetic patients and further reduce health-related quality of life (HRQoL) [6].

HRQoL is one of the most widely-used measures to self-assess the effect of the management of chronic disease on health, and monitors the physical, psychological and social aspects of personal health [7]. It is influenced by a person’s expectations, beliefs, perceptions and experiences [8]. WHOQO100, MOS-SF36 and EQ-5D are used as the generic instruments of HRQoL nowadays, while some specific instruments (e.g. EORTC and FACT-FAHI) are used according to a disease’s characteristics and symptoms [9]. In our study, we chose the EuroQoL-5 instrument (EQ-5D) to evaluate HRQol. A systematic review found that over 50 published articles had used the EQ-5D questionnaire for patients with Type 2 Diabetes Mellitus (T2DM) from 1987 to 2009, including large cross-sectional surveys, randomized clinical trials and longitudinal studies [10].

Most cross-sectional studies found that HRQoL of an individual with diabetes is worse than that of a similarly aged person without diabetes [5, 11]. However, whether HRQoL is associated with blood glucose level and HbA1c remains unclear. We enrolled 6500 participants aged 18-93 years old living in East China. Three hundred eighty six participants had T2DM. Our study was the first to assess whether blood glucose level, HbA1c and insulin resistance are predictive of changes in HRQoL.

## Methods

### Population

East China accounts for 29.2% of the population of China, recording a population of approximately 395 million people in 2011. Our study builds on the database, SPECT-China, which was a population based cross-sectional investigation in East China in 2014 involving 7200 participants living in Shanghai, Jiangsu, Zhejiang and Jiangxi [12, 13]. The registration number is ChiCTR-ECS-14005052, http://www.chictr.org.cn. Personal economic status is quite varied in East China. To decrease the disparity, we randomly chose one city with a low economic status and one city with a high status, while in rural areas we randomly chose six villages with low economic status and six villages with high economic status. The overall response rate was 73.8%. Participants who were missing lab data (*n* = 349), who refused to offer information about level of education (*n* = 1044) and marital status (491), and who were younger than 18 years old (*n* = 6), were excluded. In total, 5310 subjects were enrolled in this study. All procedures followed were in accordance with the ethical standards of the responsible committee on human experimentation (institutional and national) and with the Helsinki Declaration of 1975, as revised in 2008, and the protocol was approved by the Ethics Committee of Shanghai Ninth People’s Hospital, Shanghai Jiaotong University School of Medicine (2013(86)). Informed consent was obtained from all patients before being included in the study.

Information on confounders, e.g. gender, age, race/ethnicity, employment, marriage and location was collected via additional questionnaires. GDP per person is often considered as an indicator of the living standard of the general population [10]. We used GDP per capita in 2013 at each site to measure the economic status [14]. Hypertension and hypercholesterolemia were considered with physician diagnosis or related medication use.

### Type 2 diabetes

Diabetes cases were either self-reported, previously diagnosis by health care professionals or detected by measuring an FPG of 7.0 mmol/L or higher or an HbA1c of 6.5% or higher by American Diabetes Association criteria before the study. All of the above were confirmed via a validated supplementary questionnaire regarding blood tests, symptoms, and hypoglycemic therapy. A previous validation showed that self-reported diabetes diagnosis through supplemental questionnaire was highly accurate [15]. Newly-diagnosed diabetes cases were defined as measuring an FPG of 7.0 mmol/L or higher or an HbA1c of 6.5% or higher in our study.

### Laboratory tests

Venous blood samples were obtained after participants had fasted for at least 8 h. Fasting plasma glucose (FPG) was assessed by Beckman Coulter AU 680 analyzer (Germany) using chemiluminescence assay. Fasting insulin (FINS) was measured by the chemiluminescence method (Abbott i2000 SR, USA). In our study, we concentrated on fasting blood-glucose, and FINS used to calculate HOMA-IR = [FINS (mIU/L)]*[FPG (mmol/L)]/22.5, which assesses insulin resistance. The blood samples for the plasma glucose test were centrifuged within 1 h of collection.

### EQ-5D

To measure HRQoL, we chose the EQ-5D instrument, which was developed in 1987 by EuroQol15 research groups and is widely used in domestic studies [16]. The first part of the EQ-5D involves patients self-reporting on their health status from five perspectives: mobility (MO), self-care (SC), usual activities (UA), pain/discomfort (P/D) and anxiety/depression (A/D). Each perspective has “no problems”, “some or moderate problems” and “extreme problems” constituting a three-level scale, which scores from 1 (no problem) to 3 (extreme problems). Responses to the first part of the EQ-5D can be presented separately for each perspective in terms of a profile (EQ-5Dprofile) or converted into a weighted index (EQ-5D index) using the population preference scores of Japan [17]. The population preference scores of UK, USA and Japan are the most commonly used. Considering the absence of population preference scores for China, and the similarity of the location, geographically, racial characteristics and lifestyle between the two countries, we chose population preference scores of Japan [18]. The index ranges from −0.111 to 1, where 1 represents preferred health, 0 represents death and a score of less than 0 represents health states worse than death. The second part of the EQ-5D consists of a 20 cm visual analogue scale (VAS) with endpoints of 0, representing the worst imaginable state, and 100, representing the best. This is used to record the participant’s perception of his or her quality of life.

### Data analysis

Descriptive statistics were used to present demographic and disease-related information. Percentages and frequencies were used for the categorical variables, while for continuous variables means and standard deviations (SDs) were calculated. The data was analyzed by using the Statistical Package for Social Sciences (SPSS, version19.0). Statistical significance was set at *p* < 0.05. AVONA and t test were used to test the significance of differences among groups. Linear regression was used to test the relationship between diabetes and HRQoL. Otherwise, we used bivariate analysis to examine associations of EQ-5D scores with concomitant factors.

## Results

The study sample included 2921 men and 2989 women. The mean age of the cohort was 52.25 ± 13.41 years. A total of 95.4% were married. As shown in Table 1, less than half (26.5%) were university educated or above. Three hundred eleven participants were confirmed as type 2 diabetes mellitus. One hundred seventy nine of them were using only oral hypoglycemic agents, while 25 were using insulin only, and 13 were using both. The rest controlled their diabetes with diet and exercise.

**Table 1:** General clinical characteristics

Characteristics	Participants (*n* = 5310)
Age (mean, SD)	52.2 ± 13.4
Gender (*n*,%)	
Male	2321(43.7%)
Female	2989(56.3)
Marial status (*n*,%)	
Single	245(4.6%)
Married	5065(95.4%)
Education (*n*,%)	
Illiteracy	586(11.0%)
Primary	1084(20.4%)
Secondary	1413(26.6%)
High school	820(15.4%)
University	1266(23.8%)
Master	141(2.7%)
GDP per capita (*n*,%)	
≥15000dollars	2909(54.8%)
13,000-15000dollars	1248(23.5%)
6172-13000dollars	821(15.5%)
3000-6172dollars	332(6.3%)
Diagnosed diabetes (*n*,%)	311(5.9%)
Duration(mean, SD)	5.53(4.21)
Treatment (*n*,%^a^)	
Diet control and exercise	95(30.5%)
Oral hypoglycemic drugs only	179(57.6%)
Insulin only	25(8.0%)
Oral hypoglycemic drugs + insulin	12(3.9%)
Newly-diagnosed diabetes	296(5.5%)

Treatment %^a^ calculated as the percentage of the particular treatment in diabetes patients

In Table 2, we compared age, BMI, EQ-5D and laboratory tests in control, newly-diagnosed diabetes and diagnosed diabetes groups. There was no significant difference in age. The diabetes patients had higher values in BMI and corresponding laboratory examinations. The mean EQ-5D index and EQ-VAS scores were 0.939 ± 0.111 and 80.06 ± 11.58, respectively. The EQ-5D index and EQ-VAS of diabetes patients, was 0.922 ± 0.122 and 73.56 ± 12.71 respectively. It turned out that diabetes patients had lower EQ-5D scores than the control group and newly-diagnosed group. Otherwise, the newly-diagnosed group had a higher EQ-5D index than the control group.

**Table 2:** Characteristics of participants by diagnosis of diabetes

Variable	Non-diabetes (*n* = 4703)	Newly-diagnosed diabetes (*n* = 296)	Diagnosed diabetes (*n* = 311)	
	Mean(SD)	Mean(SD)	Mean(SD)	*p*
Age	51.50(13.55)	53.71(10.52)	54.62(1.15)	0.05
BMI	24.55(3.60)	27.83(4.52)	28.15(4.23)	0.02^*^
HbA1c	5.18(0.58)	6.67(1.48)	6.9(1.52)	<0.001^*^
FINS	37.65(27.12)	65.75(76.28)	58.31(92.22)	<0.001^*^
FPG	5.33(0.58)	7.93(2.3)	8.01(2.69)	<0.001^*^
HOMA-IR	9.04(7.18)	23.63(30.22)	20.97(32.37)	<0.001^*^
EQ-5D vas	80.06(11.58)	78.54(11.45)	75.56(12.71)	<0.001^*^
EQ-5D index	0.939(0.111)	0.94(0.112)	0.922(0.122)	0.029^*^

Data were expressed as the mean (SD)

^*^Denotes statistical significance at *P* < 0.05

**Fig. 1:**
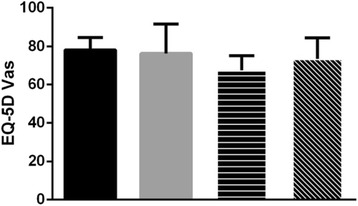
The EQ-5D Vas of different diabetes treatments.  Diet control and exercise.  Oral hypoglycemic drugs only.  Insulin only.  Oral hypoglycemic drugs and insulin. The EQ-5D VAS score of the patients using insulin only was the lowest compared to other treatments

As shown in Table 3, the EQ-5D index of males was higher than of females (p<0.001). According to our results the more developed the region, the higher the EQ-5D score. Unmarried individuals presented higher score of the EQ-5D than married individuals. As to the level of education, it turned out there was an inverted U shape curve of the EQ-5D scores, with illiteracy gaining the poorest scores, and secondary or high school education gaining the best. In diabetes patients, the economic status and level of education showed similar effects on HRQoL to the general population. In the case of the type of treatments for diabetes, the EQ-5D index of the oral hypoglycemic drugs and the insulin was lower than the other treatment groups. As for the EQ-5D VAS score, the patients using only insulin had the lowest score, while the diet and exercise group showed the highest score, which is shown in Fig. 1.

**Table 3:** Comparison of EQ-5D index and EQ-5D Vas

Variable	EQ-5D index				EQ-5D Vas			
	General population		Diabetes		General population		Diabetes	
	Mean(SD)	*p*	Mean(SD)	*p*	Mean(SD)	*p*	Mean(SD)	*p*
Gender		<0.001^*^		<0.001^*^		0.561		0.981
Male	0.95(0.11)		0.94(0.11)		79.95(11.72)		74.61(13.03)	
Female	0.93(0.11)		0.90(0.13)		80.14(11.48)		71.73(12.42)	
Marial status		<0.001^*^		0.523		0.104		0.310
Single	0.96(0.09)		0.95(0.11)		82.29(12.31)		68.33(11.69)	
Married	0.94(0.11)		0.92(0.12)		79.95(11.54)		73.67(12.73)	
Education		<0.001^*^		0.001^*^		<0.001^*^		0.668
Illiteracy	0.908(0.14)		0.860(0.14)		75.18(12.93)		69.09(14.26)	
Primary	0.935(0.11)		0.938(0.11)		78.33(11.72)		73.56(11.64)	
Secondary	0.948(0.10)		0.942(0.11)		81.58(10.99)		74.52(12.89)	
High school	0.947(0.10)		0.939(0.12)		81.39(11.48)		74.93(11.89)	
University and master	0.940(0.11)		0.925(0.12)		81.12(10.80)		75.65(11.96)	
GDP per person		<0.001^*^		<0.001^*^		<0.001^*^		0.125
≥15000dollars	0.948(0.10)		0.941(0.11)		80.15(10.96)		74.42(11.83)	
13,000-15000dollars	0.944(0.10)		0.902(0.14)		80.28(13.00)		70.86(17.67)	
6172-13000dollars	0.915(0.12)		0.880(0.14)		80.66(10.54)		74.59(11.67)	
3000-6172dollars	0.890(0.14)		0.833(0.16)		76.98(13.25)		69.46(11.97)	

Data were expressed as the mean (SD)

^*^Denotes statistical significance at *P* < 0.05

Table 4 shows the association of diabetes and EQ-5D scores. After adjusting for age, gender, BMI, GDP, marital status and level of education, it turned out that diabetes may be negatively associated with the EQ-5D Vas. Diabetes was also negatively associated with EQ-5D Index. But the relationship between diabetes and EQ-5D index decreased after adjusting for confounders.

**Table 4:** Association of diabetes and EQ-5D

	EQ-5D Index		EQ-5D VAS	
Variable	Beta	*p*	Beta	*p*
Diabetes	−0.017	0.008*	−0.609	<0.001*
Model 1	−0.007	0.263	−0.470	<0.001*
Model 2	−0.011	0.086	−0.480	<0.001*

Model 1 adjusted for age, gender

Model 2 adjusted for age, gender, BMI, GDP, marital status and level of education

*Denotes statistical significance at *P* < 0.05

Table 5 shows the correlation between EQ-5D and age. The older the person was, the lower the EQ-5D Vas and EQ-5D index were. We also found that the EQ-5D Vas of the population was adversely associated with FPG, HbA1c and HOMA-IR, while the EQ-5D index was not quite associated with them, both in diabetes patients and non-diabetes patients. Otherwise, the EQ-5D index of diabetes was positively correlated with FPG. However, the correlation was weak according to our results.

**Table 5:** Relevance in EQ-5D index and EQ-5D Vas

	Age	*p*	FPG	*p*	HbA1c	*p*	HOMA-IR	*p*
EQ-5D index of population	−0.121	<0.001^*^	−0.001	0.930	−0.022	0.114	0.007	0.604
EQ-5D index of diabetes	−0.2	<0.001^*^	0.120	0.035^*^	0.073	0.200	0.011	0.848
EQ-5D vas of population	−0.268	<0.001^*^	−0.128	<0.001^*^	−0.116	<0.001^*^	−0.070	<0.001*
EQ-5D vas of diabetes	−0.144	0.011^*^	−0.011	0.853	−0.042	0.458	−0.141	0.013*

Data were value of Pearson’s r

*FPG* fasting plasma glucose, *HbA1c* glucosylated hemoglobin, *HOMA-IR* homeostasis model assessment of insulin resistance

^*^Denotes statistical significance at *P* < 0.05

## Discussion

HRQoL is one of the most important measures used to assess the effect of the management of chronic diseases on health. To our knowledge, this was the first study to use the EQ-5D instrument to investigate the HRQoL in the East China population, including in patients with type 2 diabetes. The qualitative findings of this study of 5310 participants provide a more thorough understanding of the performance of the EQ-5D in China. First of all, there was a significant difference in HRQoL by gender. The mean score of the EQ-5D index was lower in females compared to males, which is similar to the finding of other studies [19–21]. This difference could be due to lifestyle behavior differences between men and women in society as women normally spend more time indoors doing housework [22]; this could lead to less physical activity and bad diet. Otherwise, women are more likely to have depression or anxiety. Previous studies revealed that age, marital status, level of educational and economic status were all significantly associated with EQ-5D [23]. According to our results, the EQ-5D scores were much lower in the less economically developed regions. The majority of the participants in the less developed regions are doing heavy labor or farming in their daily lives, which causes pain and discomfort. A lack of income leading to depression and inadequate medical care might be another reason. Unmarried individuals present higher scores of the EQ-5D, which might not be meaningful because the young unmarried might have the most active and healthy status leading to high EQ-5D scores. While the level of education turned out an inverted U shape curve of the EQ-5D scores, with illiteracy gaining the poorest scores and secondary or high school gaining the best scores. The pursuit of a healthier lifestyle, along with efforts to take better care of themselves, might cause anxiety and psychoneurosis for well educated people. The poorly educated population often come from the poorly developed regions. A lack of knowledge might keep them from appropriate health services. In addition, misunderstanding of the questionnaire and casual answers cannot be ruled out. After all, our study demonstrates the general situation of the HRQoL of the population in East China, which is associated with varied socio-demographic characteristics.

In our study, not only did we calculate the EQ-5D scores and assess the socio-demographic characteristics correlated with HRQoL and the relationships between the EQ-5D Vas and scores, but we also evaluated whether the blood glucose level, the HbA1c, the insulin resistance and the type of treatment were associated with HRQoL. Previous studies have shown that the EQ-5D has been used to measure HRQoL of diabetes patients [24–26]. It is widely found from previous studies that type 2 diabetes patients have moderately lower scores of HRQoL than the general population of similar age [25, 27], which is similarly demonstrated in our study, where the mean EQ-5D index and EQ-VAS scores of diabetes patients were 0.922 ± 0.122 and 73.56 ± 12.71, respectively, compared to 0.939 ± 0.111 and 80.06 ± 11.58 for non-diabetes patients. It stands to reason that the socio-demographic characteristics influence consistently the EQ-5D scores of diabetes patients including gender, economic development of region and marital status. After adjusting for the confounds including age, gender, BMI, GDP, marital status and level of education, diabetes may be negatively associated with EQ-5D Vas while it may not be associated with the EQ-5D index. Diabetes patients, especially newly-diagnosed patients, may not have much trouble in mobility, self-care and everyday activities. But suffering from diabetes does affect their assessment of their health and quality of life. In our results, the newly-diagnosed group had similar EQ-5D vas and even higher EQ-5D index than the control group. Most newly-diagnosed patients in our study had hyperglycemia without corresponding symptoms including polyuria, polydipsia and weight loss; it may not affect their health-related quality of life yet. Regarding the treatment group, the patients who used insulin injection only, presented the lowest EQ-5D Vas. Polonsky WH et al. found that the emotional distress could be caused by the worries about hypoglycemia and poor glycemic control [28]. Otherwise, it might be also explained by the pain of multi-injection of insulin.

Several previous studies reported that increased age was associated with lower HRQoL [14, 29]. We also found that age is quite adversely associated with the EQ-5D index and EQ-5D Vas. It might be assumed that aged patients couldn’t take care of themselves and experience pain but are still satisfied with their current status. Nevertheless, there was no significant difference of the EQ-5D scores between patients with normal HbA1c and abnormal HbA1c; and neither with HOMA-IR and FPG. We also found almost no linear correlation between the EQ-5D index and tests results, while the FPG, the HbA1c and the HOMA-IR was weakly correlated with the EQ-5D Vas score. According to our results, only the EQ-5D Vas had relevance with the laboratory tests. EQ-5D Vas stands for one’s self-assessment of health status. Bazelmans E et al. found that self- reported previous anxiety was more prevalent in the control group of diabetes patients [30, 31]. The weak correlation might be explained by self-anxiety. Since there is less literature paying attention to this point directly, we couldn’t compare the results more objectively. However, as the EQ-5D is used as the one of the most important instruments to assess HRQoL, we should not only stick to the laboratory tests results. The EQ-5D is more like a macroscopic index focusing on one’s entire state of health, including pain and the emotion. When one’s laboratory results augment or decrease, the patient might not feel poorly or in pain. Hence, the abnormality of laboratory tests may not have direct influence on the EQ-5D. However, the high HbA1c and FPG usually account for the poor control of diabetes, which may lead to complications including stroke, heart failure, myocardial infarction, ischemic heart disease, renal failure, blindness and amputation [32]. Hayes A et el. found that the common complications of diabetes significantly reduce health-related quality of life measured by the EQ-5D [33]. In the future, we will arrange a follow-up with the same population in a couple of years exploring whether the HbA1c, FPG and HOMA-IR are correlated with the change of HRQoL. After all, the primary target of decreasing FPG and controlling HbA1c is still consistent.

The sample size, cross-sectional design and repeated measurement enabled examination of HRQoL, in both the non-diabetes and diabetes patients, and are important strengths of our study. Observational research cannot establish causality. An additional limitation was that the questionnaire was conducted in such a limited time that the participants might have completed the EQ-5D Vas without understanding thoroughly the meaning of the endpoints, especially the poorly-educated participants and the more elderly ones.

## Conclusion

The study has highlighted that the overall health-related quality of life of the population in East China is moderate. Diabetes patients had a low score of health-related quality of life. Health-related quality of life was associated with age, gender, level of education, economic development of the region and marital status. Increased age resulted in lower EQ-5D scores. The EQ-5D Vas was weakly adversely associated with FPG, HbA1c and HOMA-IR. This is the first report about HRQoL in East China including diabetes patients, and can be taken into account by healthcare professionals when planning holistic patient treatment.

## Abbreviations

DM: Diabetes mellitus
EQ-5D: EuroQoL-5 dimension
FPG: Fasting plasma glucose
HbA1c: Glycosylated hemoglobin
HRQoL: Health-related quality of life
IDF: International diabetes federation
